# Supplementary material for: Regulation of the EphA2 receptor intracellular region by phosphomimetic negative charges in the kinase-SAM linker
Source: Nat Commun. 2021 Dec 2;12:7047. doi: 10.1038/s41467-021-27343-z (PMC8639986; doi:10.1038/s41467-021-27343-z)
Supplement: Supplementary file 1 — Supplementary Information [file 41467_2021_27343_MOESM1_ESM.pdf]

Supplementary Information

Regulation of the EphA2 Receptor Intracellular Region by Phosphomimetic Negative Charges in the Kinase-SAM Linker

Bernhard C. Lechtenberg[1,2,3]*, Marina P. Gehring[1], Taylor P. Light[4], Christopher R. Horne[2,3], Mike W. Matsumoto[1], Kalina Hristova[4] and Elena B. Pasquale[1]*

[1] Cancer Center, Sanford Burnham Prebys Medical Discovery Institute, La Jolla CA 92037, USA

[2] Ubiquitin Signalling Division, The Walter and Eliza Hall Institute of Medical Research, Parkville, Victoria 3052, Australia

[3]Department of Medical Biology, The University of Melbourne, Parkville, Victoria 3010, Australia

[4] Department of Materials Science and Engineering, Institute for NanoBioTechnology, Johns Hopkins University, 3400 Charles Street, Baltimore, MD 21218

* Correspondence: lechtenberg.b@wehi.edu.au and elenap@sbpdiscovery.org

Supplementary Table 1. Data collection and refinement statistics

	EphA2 WT	EphA2 S897E/S901E	EphA2 S901E
**Data collection**			
Space group	I 1 2 1	P 3_2 2 1	I 1 2 1
Cell dimensions			
a, *b*, *c* (Å)	123.1, 55.0, 135.7	94.5, 94.5, 99.7	121.6, 54.6, 135.5
α, β, γ (°)	90, 94.86, 90	90, 90, 120	90, 95.2, 90
Resolution (Å)	20.49–1.75(1.78–1.75) *	29.53–2.80(2.95–2.80)	29.25–2.30(2.38–2.30)
R_{sym} or R_{merge}	0.059(0.690)	0.285(1.956)	0.089(0.656)
$I / \sigma I$	13.4(1.4)	10.2(1.4)	12.0(1.8)
Completeness (%)	99.9(100.0)	99.9(100.0)	99.8(99.8)
Redundancy	3.6(3.5)	10.9(11.0)	3.7(3.7)
**Refinement**			
Resolution (Å)	20.48–1.75(1.81–1.75)	29.53–2.80 (2.90–2.80)	29.25–2.30(2.38–2.30)
No. reflections	90866 (9053)	13027 (1269)	39703 (3948)
R_{work} / R_{free}	0.1883/0.2173	0.1976/0.2461	0.1884/0.2257
No. atoms	6472	2913	6277
Protein	5764	2882	5847
Ligand/ion	62	1	62
Water/solvent	646	30	368
B-factors			
Protein	32.3	56.8	42.9
Ligand/ion	41.9	121.1	51.5
Water/solvent	35.1	36.9	38.9
R.m.s. deviations			
Bond lengths (Å)	0.004	0.003	0.002
Bond angles (°)	0.71	0.50	0.52

Data for each structure were collected from a single crystal.
*Values in parentheses are for highest-resolution shell.

Supplementary Table 2: SAXS data collection and analysis statistics

**Data collection parameters**					
Instrument	Australian Synchrotron SAXS/WAXS beamline				
Detector	PILATUS3-2M (Dectris)				
Detector distance (m)	3				
Wavelength (Å)	1.0332				
Total q range (Å^{-1})	0.005 – 0.5				
Maximum flux at sample	8 x 10^{12} photons per second at 12 keV				
Exposure time	Continuous 1 second frame measurements				
Sample configuration	SEC-SAXS with co-flow				
Temperature	12				
**Software employed**					
Primary data reduction	ScatterBrain (Australian Synchrotron)				
Data processing	PRIMUS (ATSAS)				
**Analysis statistics**	**WT**	**S892E**	**S897E**	**3E**	**5E**
$I(0)$ (cm^{-1}) (from Guinier analysis)	0.026 ± 9.8e-5	0.027 ± 1.4e-4	0.026 ± 1.0e-4	0.027 ± 1.3e-4	0.029 ± 1.5e-4
R_g (Å) (from Guinier analysis)	25.87 ± 0.16	28.04 ± 0.25	26.35 ± 0.17	27.29 ± 0.23	31.57 ± 0.25
R_g (Å) (from $P(r)$ analysis)	26.49 ± 0.03	28.99 ± 0.03	27.13 ± 0.03	28.10 ± 0.03	32.84 ± 0.03
D_{max} (Å)	104	116	104	104	123
Porod volume estimate (Å^3)	65327	69929	65852	69060	81873
**Molecular mass (MM) determination**					
MM (from Porod Volume, kDa)	38.4	41.1	38.7	40.6	48.2
MM (from SAXSMoW2*, $q_m = 8/R_g$, kDa)	47.6	53.6	48.9	51.1	65.4
Calculated monomer MM from sequence (kDa)	47.2	47.2	47.2	47.2	47.2

*http://saxs.ifsc.usp.br/

Supplementary Table 3. Kinase phosphorylation of EphA2 linker peptide with pS897

Protein Kinase	CPM	Protein Kinase	CPM	Protein Kinase	CPM	Protein Kinase	CPM
PKC zeta	192,007	PDK1	10,417	MEKK2	4,367	MARK1	2,197
PAK3	151,679	CAMKK1	10,287	CK2 alpha 2	4,254	MNK1	2,187
CK1 gamma 1	139,024	AMPK (A1/B2/G2)	10,227	MLK4	4,176	MEKK6	2,178
NEK7	119,908	ZAK	10,208	GSK3 alpha	4,094	STK21(CIT)	2,124
PKC beta I	115,018	NUAK1	10,059	CLK1	4,068	TAK1-TAB1	2,080
PKC beta II	111,980	ALK6 (BMPR1B)	10,006	HIPK3	4,009	ERK5	2,063
PKC gamma	108,805	TSSK2	9,883	VRK1	3,923	DRAK2 (STK17B)	1,980
TBK1	106,427	DAPK3	9,822	AMPK (A2/B2/G3)	3,912	CLK3	1,958
PKC alpha	102,845	AURORA C	9,740	RSK4	3,846	MRCK alpha	1,936
NEK2	99,876	CAMK2 gamma	9,676	BRAF	3,826	GRK6	1,928
MLK1	98,444	VRK2	9,550	CDK2/CyclinA1	3,817	IKK alpha	1,852
PKC eta	95,137	CHK1	9,447	BRSK1	3,800	NEK8	1,844
PKC theta	82,115	STK33	9,190	CASK	3,793	PCTK3 (CDK18)	1,812
PKC iota	75,819	ALK1	8,852	EEF2K	3,739	DYRK4	1,805
AURORA B	72,966	PHKG2	8,761	MST4	3,722	PFTK1(CDK14)Cyc	1,776
CAMK2 beta	71,807	CAMK1 beta	8,751	SNRK	3,679	SIK	1,724
NEK6	67,524	CDK5/p25	8,524	CAMK1	3,657	MEKK1	1,691
ULK1	65,909	MSK1	8,483	MARK3	3,624	PEAK1	1,680
ULK2	64,661	PRKG1	8,362	PCTK2 (CDK17)	3,596	PKC nu	1,680
PKC delta	59,375	PRKG2	8,340	CDK5/p35	3,590	PDHK1	1,668
CK1 alpha 1	58,763	TAOK1	8,257	NLK	3,584	MST3	1,645
CDC7/DBF4	55,210	TGFBR2	8,047	MYLK3	3,583	NDR	1,637
CK1 gamma 3	54,848	NIM1	7,945	CAMK1 delta	3,539	SRPK2	1,632
PKAc beta	51,041	PAK7	7,916	MLCK	3,528	RAF1(EE)	1,607
CK1 alpha 1L	45,567	MEKK3	7,799	RIPK1	3,521	CDK2/CyclinO	1,586
CK1 gamma 2	40,800	IKK beta	7,760	STK3	3,499	p38 gamma	1,576
SGK3	40,181	RSK2	7,721	MYLK4	3,498	ERK2	1,570
MST1	38,378	p70S6K	7,590	HIPK4	3,469	LATS2	1,566
TTBK2	35,044	CAMKK2	7,398	CLK2	3,416	GRK7	1,543
PKAc alpha	34,771	TLK2	7,236	CAMK1 gamma	3,353	MEK5	1,504
TLK1	34,750	AMPK (A2/B2/G1)	7,235	BRSK2	3,331	JNK1	1,491
TTBK1	34,299	DAPK1	7,166	CDK2/CyclinE1	3,316	HUNK	1,471
EIF2AK3	32,945	AMPK (A1/B2/G1)	6,987	MAPKAPK2	3,270	EIF2AK2	1,429
PKC epsilon	32,376	PKD2	6,956	AKT3	3,269	p70S6Kb	1,419
KDR	32,100	AKT1	6,708	MAK	3,166	MEK6	1,362
GCK	28,599	NIK	6,688	PIM1	3,137	PLK2	1,312
NEK9	27,568	WNK1	6,613	FASTK	3,133	CDK6/CyclinD1	1,283
PKAc gamma	27,509	MAPKAPK5	6,561	BUB1B	3,121	GSK3 beta	1,281
PAK1	27,316	MARK4	6,474	COT	3,092	KSR1	1,192
AMPK (A1/B1/G2)	24,259	CDK1/CyclinA2	6,408	CDK9/CyclinK	3,040	MNK2	1,186
CK1 delta	23,576	MYO3 alpha	6,379	SRPK1	3,030	GRK3	1,154
YSK4	23,510	CAMK2 delta	6,359	ERN1 (IRE1)	3,002	PDHK4	1,153
ULK3	21,762	KHS1	6,222	MAPKAPK3	2,979	SIK3	1,123
NEK5	20,936	CDK1/CyclinA1	5,961	p38 alpha	2,945	IKK epsilon	1,109
TAOK3	20,457	ALK2	5,902	TXK	2,945	NDR2 (STK38L)	1,104
EIF2AK4 (GCN2)	17,969	ALK4	5,897	RIPK5	2,930	DMPK	1,103
DCAMKL2	17,207	AKT2	5,830	ROCK1	2,884	MSK2	1,103
CK1 epsilon	16,577	HGK	5,774	PAK6	2,861	Haspin (GSG2)	1,096
CDK9/CyclinT2	15,002	PDHK3	5,753	DYRK3	2,847	PLK3	1,057
RIPK3	14,926	AMPK (A2/B1/G3)	5,687	HIPK2	2,842	PAK2	1,036
HPK1	14,096	p38 beta	5,478	PIM3	2,757	PDHK2	1,036
STK32B(YANK2)	13,961	TAOK2	5,418	SBK1	2,745	ERK1	1,033
AURORA A	13,527	DRAK1 (STK17A)	5,371	NUAK2	2,665	MEK1	1,031
AMPK (A1/B1/G3)	13,405	GRK1	5,206	CDK7/CyclinH1/MN/	2,662	SGK1	1,025
DCAMKL1	13,380	ALK3 (BMPR1A)	5,101	PIM2	2,623	MEK2	1,009
MYLK2	13,209	CHK2	5,081	DYRK1a	2,620	CDK3/CyclinE1	1,000
PLK4	12,525	AMPK (A1/B2/G3)	5,073	p38 delta	2,614	TESK2	973
DYRK2	12,429	PAK4-GTP	5,035	PASK	2,590	TGFBR1 (ALK5)	959
TTK	12,279	HIPK1	5,000	SGK2	2,588	NEK1	949
BMPR2	11,689	CLK4	4,997	TSSK1B	2,578	LOK	910
CAMK2 alpha	11,654	DAPK2	4,974	MLK3	2,548	IRAK2	899
NEK11	11,639	TNIK	4,961	MELK	2,533	LRRK2	899
CK2 alpha 1	11,509	ERN2 (IRE2)	4,930	PHKG1	2,490	PKN3/PRK3	892
RSK3	11,244	ASK1	4,850	GRK5	2,471	JNK3	860
ROCK2	11,187	IRAK4	4,848	PLK1	2,467	GLK	818
STK39 (STLK3)	10,975	PKC mu	4,839	GRK2	2,427	PRKX	802
AMPK (A2/B1/G2)	10,972	CDK6/CyclinD3	4,781	RIPK2	2,371	LATS1	797
AMPK (A2/B1/G1)	10,963	SLK	4,666	QIK	2,354	STK36	794
PKN2/PRK2	10,961	TOPK	4,627	EIF2AK1 (HRI)	2,353	STK19	793
RSK1	10,918	CAMK4	4,553	MINK1	2,318	JNK2	751
NEK3	10,845	MYO3 beta	4,527	MSSK1	2,248	CDK4/CyclinD1	745
AMPK (A2/B2/G2)	10,827	CDK2/CyclinA2	4,455	ICK	2,241	STK32C (YANK3)	685
AMPK (A1/B1/G1)	10,681	MARK2	4,441	KSR2	2,236	PKN1/PRK1	51
MLK2	10,481	NEK4	4,413	PCTK1(CDK16)Cyc	2,226		
SOK1	10,460	MRCK beta	4,372	LIMK1	2,221		

> 100,000
50,000 - 99,999
25,000 - 49,999
10,000 - 24,999

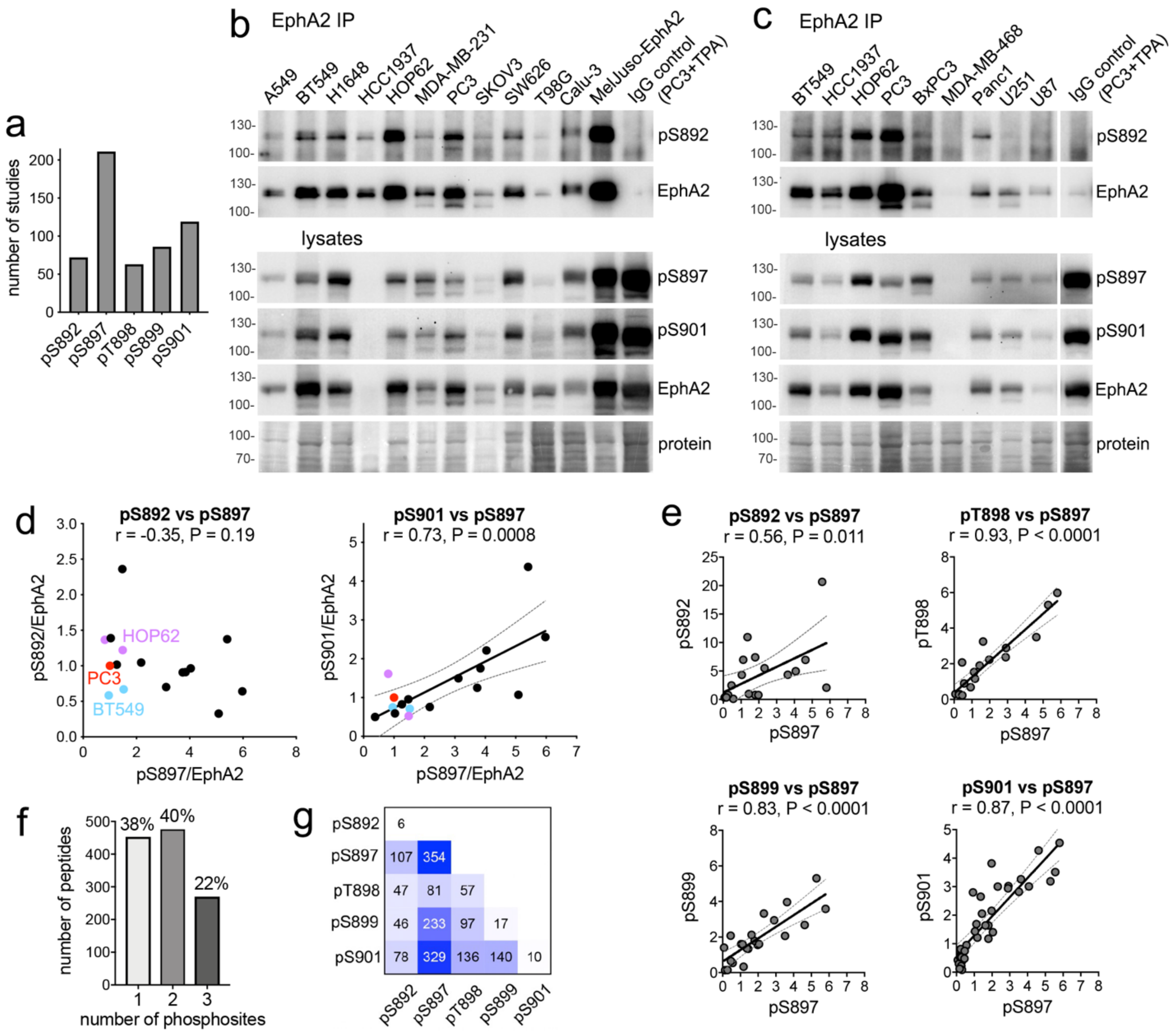

Supplementary Figure 1. EphA2 is phosphorylated on multiple serine/threonine residues in the kinase-SAM linker in cancer cell lines. (**a**) Number of studies identifying the indicated phosphosites. The higher number of studies identifying phosphorylated S897 may be due to the many mass spectrometry experiments in which peptides were purified for analysis using phospho-AKT substrate antibodies, which recognize the phosphorylated S897 motif. Data are from the 2021 PhosphoSite database (phosphosite.org). (**b, c**) Cancer cell lines were screened in two experiments for EphA2 phosphorylation on S892, S897 and S901. EphA2 immunoprecipitates were probed with an antibody specific for pS892 and reprobed for EphA2. Lysates were probed in separate blots with antibodies specific for pS897 or pS901 and reprobed for EphA2. Amido black staining shows relative protein loading in different lanes. The white vertical line in c indicates removal of an irrelevant lane. Molecular weight markers, in kDa, are indicated. (**d**) Correlation of pS892 or pS901 versus pS897, normalized to total EphA2 levels, from the blots in b and c. The values in each blot were normalized to the value for PC3 cells (in red) measured in the same blot. Duplicate measurements for BT549 cells are shown in blue and for HOP62 cells in purple. (**e**) The number of peptides containing each phosphosite, identified by mass spectrometry in lung and breast cancer cell lines, were normalized to the number of peptides containing the same phosphorylation site in a reference pool of 16 non-small cell lung cancer cell lines. The original data used for these analyses are from Supplemental Table S2 in ref.[1]. In d and e, the Pearson correlation coefficient, r, and the P value (two-tailed) for the significance of the correlation are shown. The best fit interpolation line (black line) and 95% confidence interval (grey dotted lines) are shown in the panels with significant correlation. (**f**) Sixty-two % of the phosphopeptides

from the EphA2 linker contain multiple phosphosites. Data are from the 2014 PhosphoSite database. (**g**) Different representation of the data in f, showing the number of peptides in which the indicated phosphosites were detected together. Prism software (GraphPad) was used to generate graphs and for statistical analysis. Source data for b, c, d are provided in the Source Data file.

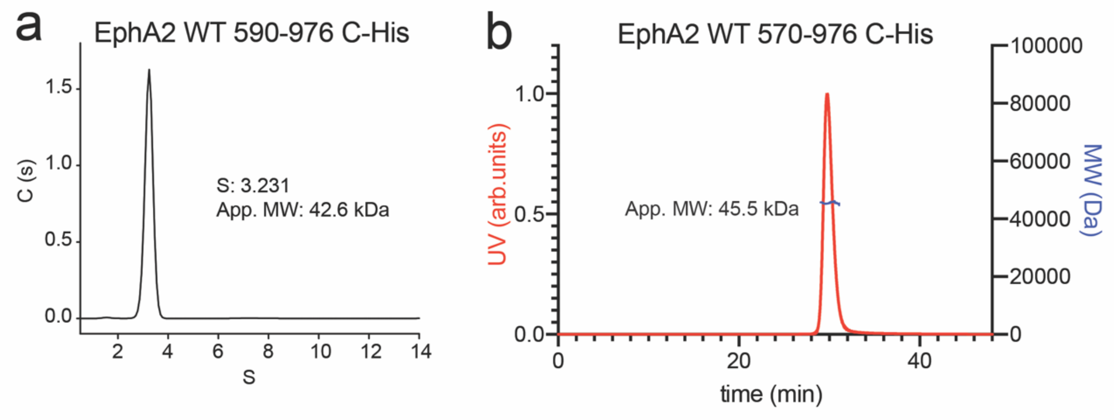

Supplementary Figure 2. The EphA2 intracellular region is monomeric in solution. (**a**) Sedimentation velocity measured by analytical ultracentrifugation for the EphA2 WT intracellular region (residues D590-I976) used for crystallization, shows that it is a monomer with an apparent molecular weight of 42.6 kDa, which closely matches the expected molecular weight of 43.9 kDa. (**b**) Size-exclusion chromatography coupled to multi-angle light scattering (SEC-MALS) for the EphA2 WT intracellular region (residues S570-I976) used in SEC-SAXS and HDX-MS experiments, shows that it is a monomer with an apparent molecular weight of 45.5 kDa, closely matching the expected molecular weight of 47.2 kDa. Source data for a, b are provided in the Source Data file.

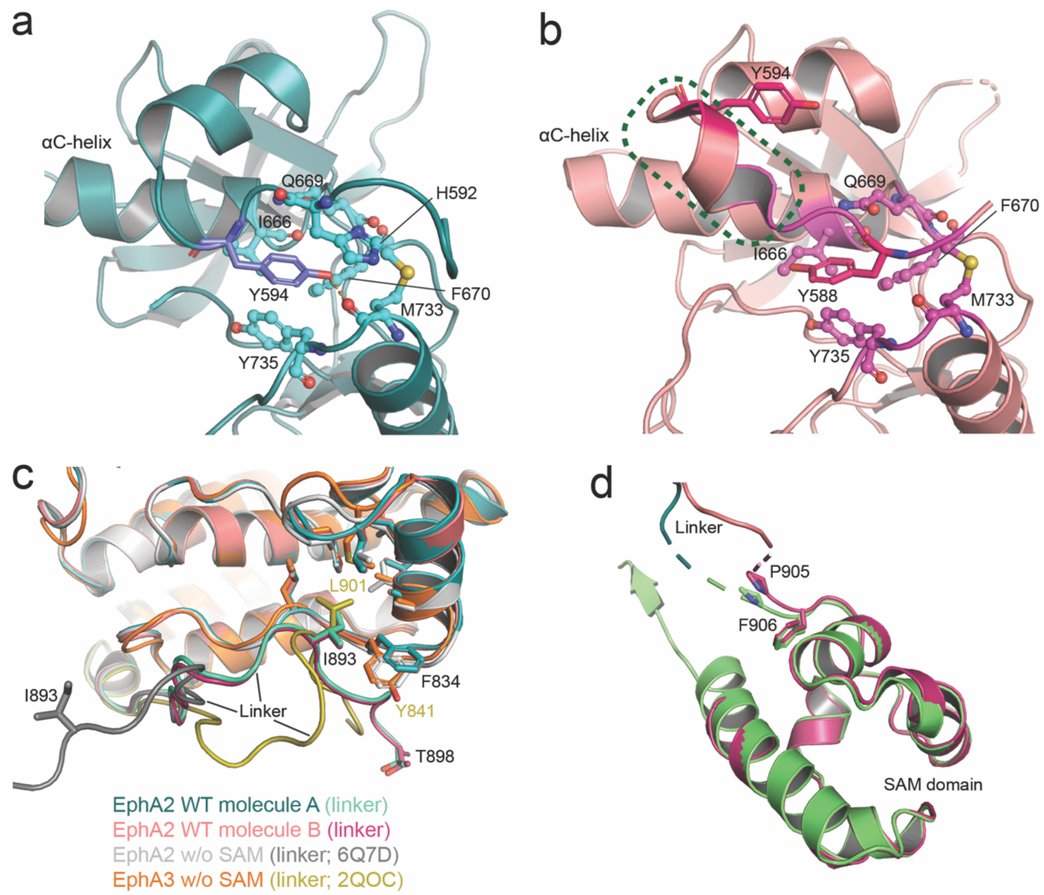

Supplementary Figure 3. Molecular details of the interaction between the non-phosphorylated EphA2 juxtamembrane segment and the kinase domain. (**a**) In our structure (PDB 7KJA [http://doi.org/10.2210/pdb7KJA/pdb]), the EphA2 juxtamembrane segment (molecule A shown) forms a short, 2-turn helix comprising residues P597–F604. Y594 is surrounded by a hydrophobic pocket lined by I666 and F670 at the end of the αC-helix and residues M733 and Y735, and additionally forms a hydrogen bond with the backbone of M733 (dashed line). (**b**) In a previous structure of the EphA2 juxtamembrane segment and kinase domain (residues K586-I875; PDB 5EK7[2] [http://doi.org/10.2210/pdb5EK7/pdb]), Y588 rather than Y594 binds to the same hydrophobic pocket. In this previous structure, the juxtamembrane segment forms an additional short helix (outlined by a dotted green line) leading to the observed differences. (**c**) Superposition of molecules A and B of our EphA2 WT structure (colors as in Fig. 1), an EphA2 kinase domain structure (residues A599-P896; PDB 6Q7D [http://doi.org/10.2210/pdb6Q7D/pdb], grey) and an EphA3 kinase domain (PDB 2QOC[3] [http://doi.org/10.2210/pdb2QOC/pdb], orange). Linker residues I875-T898 of the two molecules in our EphA2 structure have the same conformation, which differs from the linker conformation of EphA2 without the SAM domain, whereas some features (N-terminal part of the linker and interactions of the I893 hydrophobic residue) resemble the linker conformation of EphA3 without the SAM domain. Kinase domain residues forming a hydrophobic pocket that accommodates EphA2 I893 or EphA3 L901 are shown as sticks. (**d**) The two molecules in our EphA2 WT structure were superposed based on their SAM domains. The first residue of the SAM domain (F906) has the same position in the two EphA2 molecules, whereas differences are observed for the first residue after the missing portion of the linker (P905). Colors as in Fig. 1.

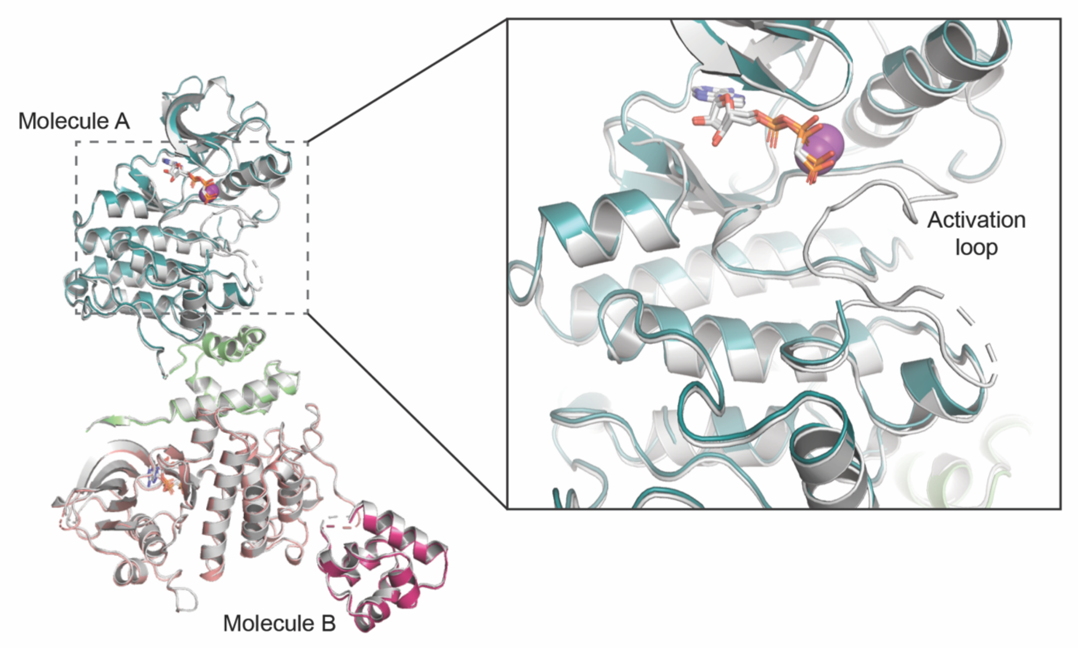

Supplementary Figure 4. Crystal structure of the intracellular region of the EphA2 S901E mutant. Superposition of the structures of the intracellular regions of EphA2 WT (colored as in Fig. 1) and the S901E mutant (grey, resolution 2.3 Å). The two structures are almost identical, with an overall RMSD calculated by aligning all Cα atoms of 0.48 Å. The RMSD obtained by aligning the individual molecules is 0.37 Å for molecule A and 0.34 Å for molecule B. A notable difference between the two structures is that most of the activation loop (residues L760-I779) is undefined in the EphA2 WT structure (the undefined region of the loop is not shown), whereas only a small portion of the activation loop (T773-G777) is undefined in molecule A of the EphA2 S901E mutant structure (indicated by a dashed line). This difference is possibly due to the slightly smaller unit cell dimensions and tighter crystal packing of the S901E mutant. The inset shows an enlargement of the indicated portion of the structures in a slightly tilted orientation. AMPPCP is shown as sticks, Mg^{2+}-ions as purple spheres.

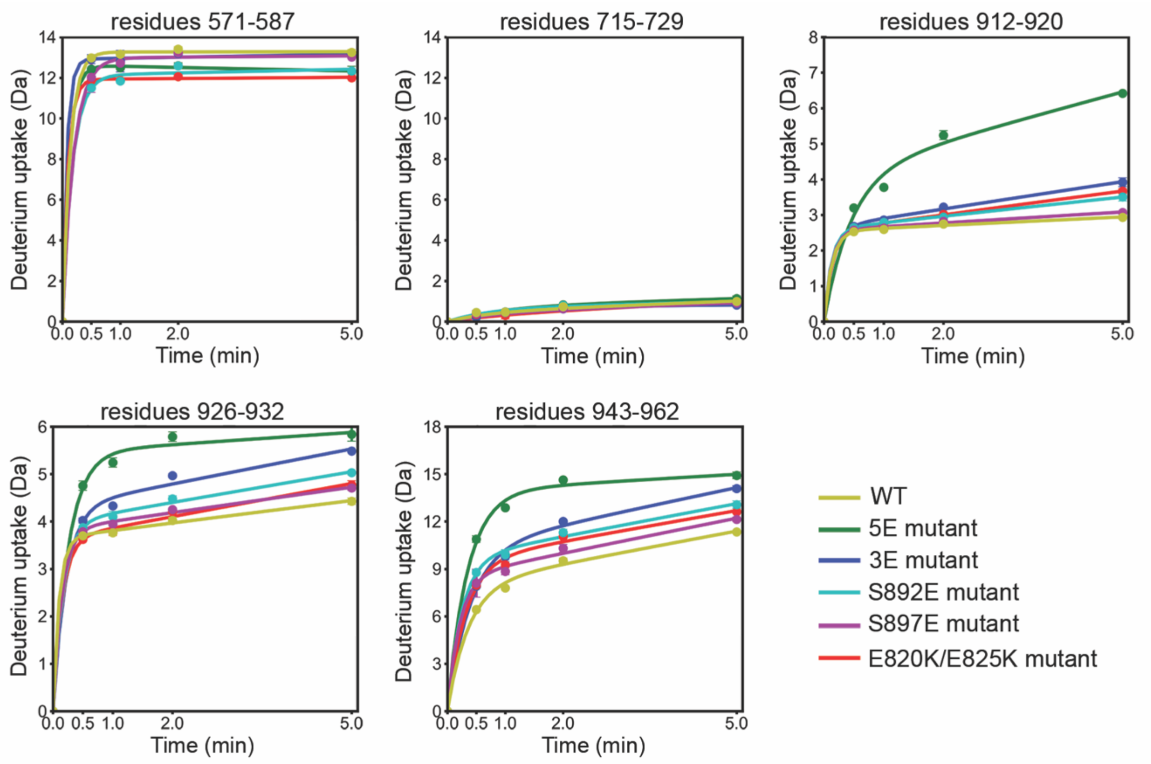

Supplementary Figure 5. Time course of hydrogen-deuterium exchange for key peptides. Deuterium uptake in the indicated peptides is plotted against time. The Y-axes are scaled to the maximum theoretically possible deuterium uptake for each peptide. Colors denote different mutants, as indicated. Averages +/- SEM from 3 technical replicates are shown. Source data are provided in the Source Data file.

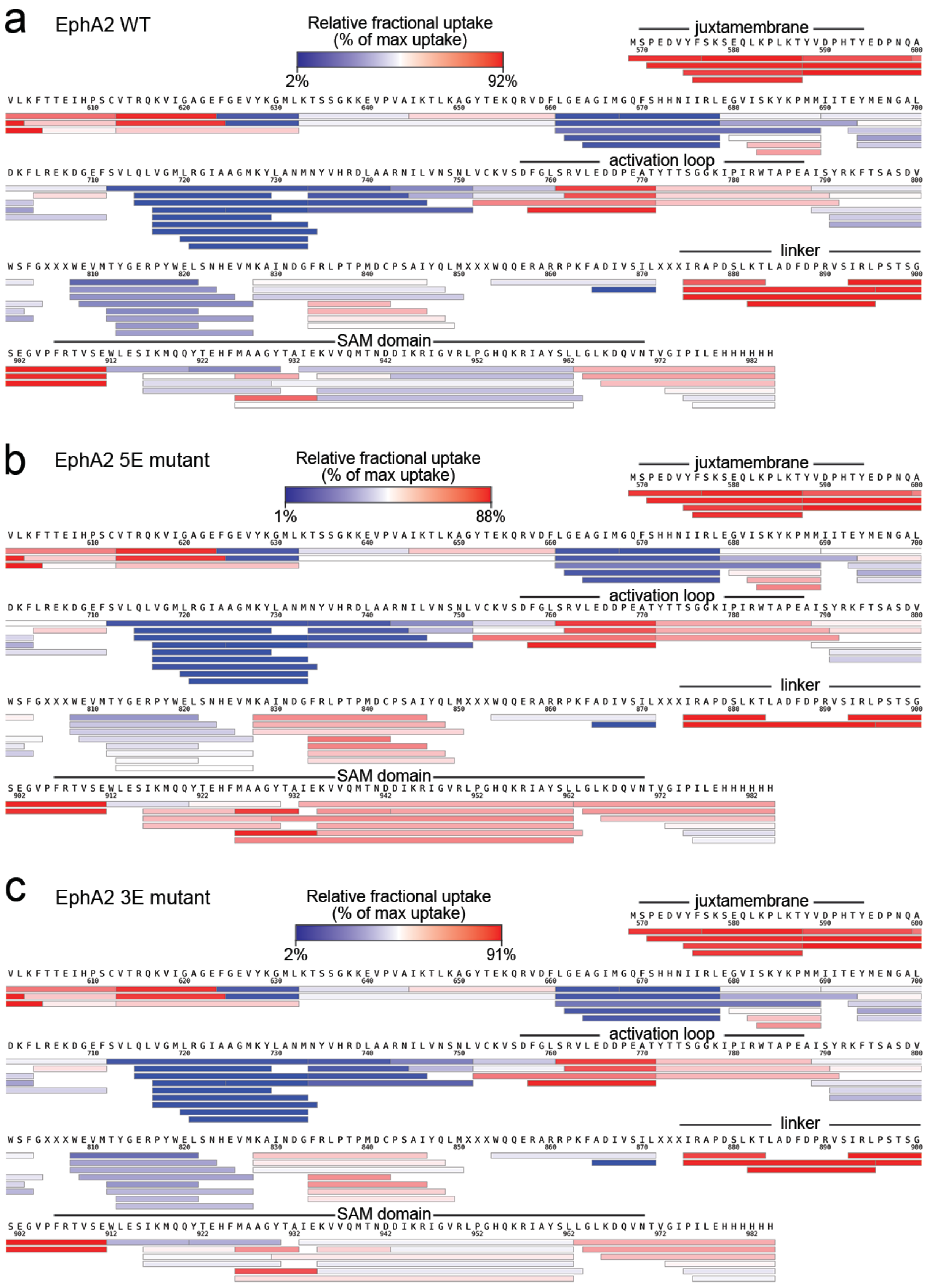
a
EphA2 WT
Relative fractional uptake
(% of max uptake)
2%
92%
juxtamembrane
activation loop
linker
SAM domain
b
EphA2 5E mutant
Relative fractional uptake
(% of max uptake)
1%
88%
juxtamembrane
activation loop
linker
SAM domain
c
EphA2 3E mutant
Relative fractional uptake
(% of max uptake)
2%
91%
juxtamembrane
activation loop
linker
SAM domain

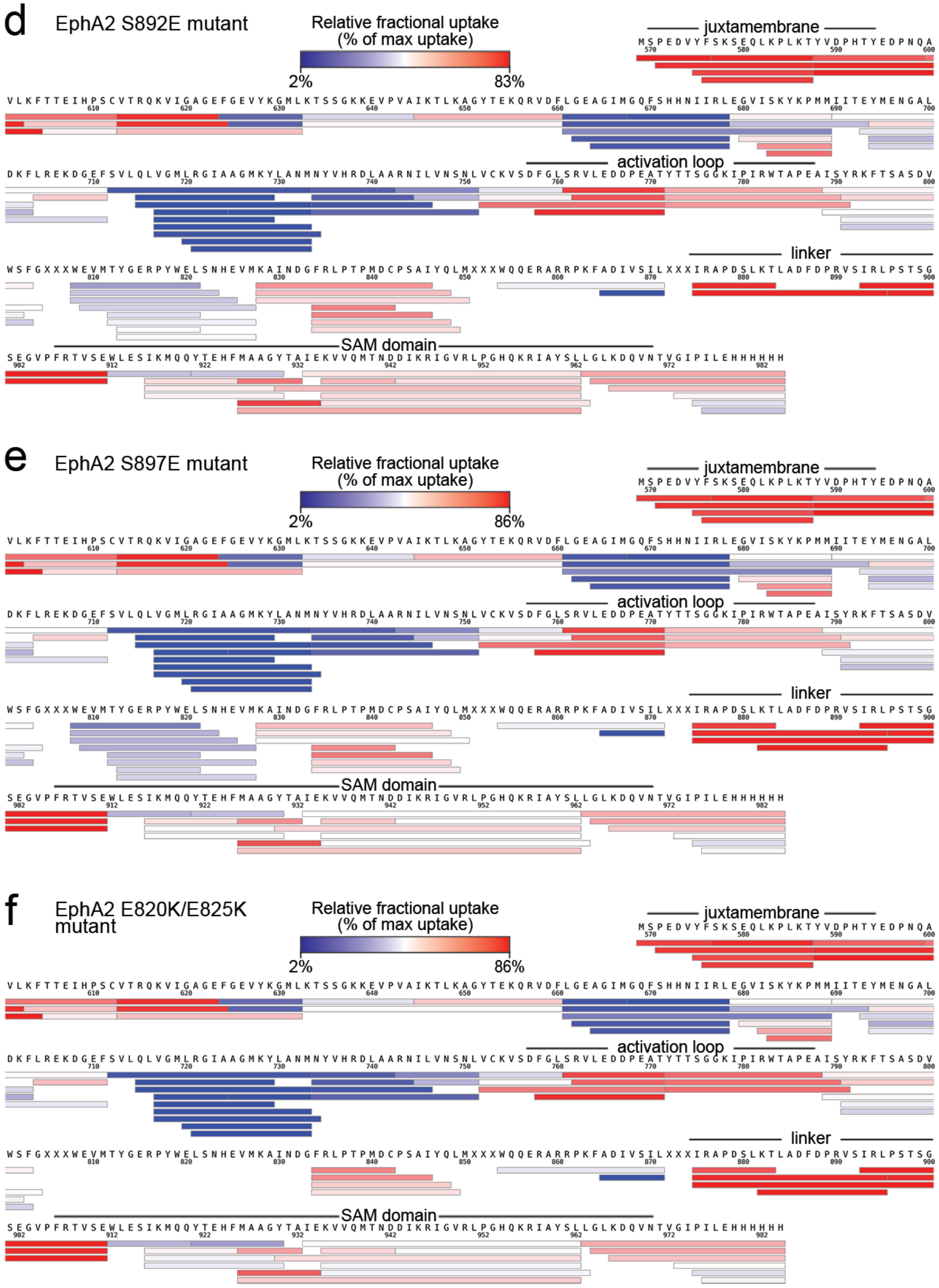

Supplementary Figure 6. Coverage plots from HDX-MS experiments and relative fractional uptake after 0.5 min deuterium exposure. (**a**) EphA2 WT (**b**) 5E mutant (**c**) 3E mutant (**d**) S892E mutant (**e**)

S897E mutant (**f**) E820K/E825K mutant. In six experiments, we assigned 111 peptides covering 97.8% of the EphA2 WT sequence with 4.0 average redundancy. Data represent averages from n = 3 successful technical replicates for each mutant. Source data are provided in the Source Data file.

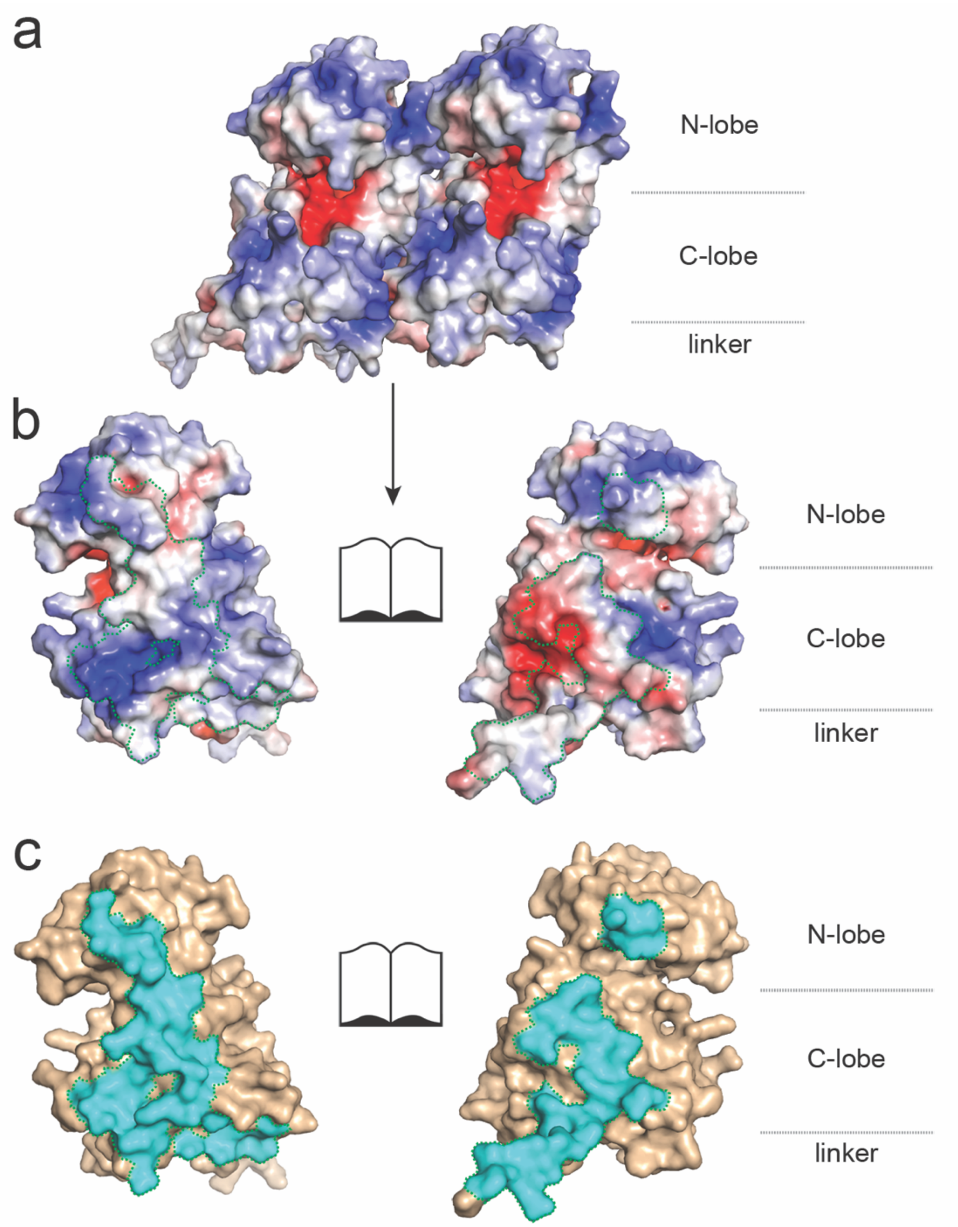

Supplementary Figure 7. Putative EphA2 dimer. (**a**) Crystallographic EphA2 kinase domain dimer (PDB 6FNG [http://doi.org/10.2210/pdb6FNG/pdb])[4] with electrostatic surface. (**b**) Open book view of the dimer shown in A. The dimer interfaces are outlined by green dotted lines. (**c**) View as in B, both molecules are colored in wheat with dimer interfaces shown in cyan.

a

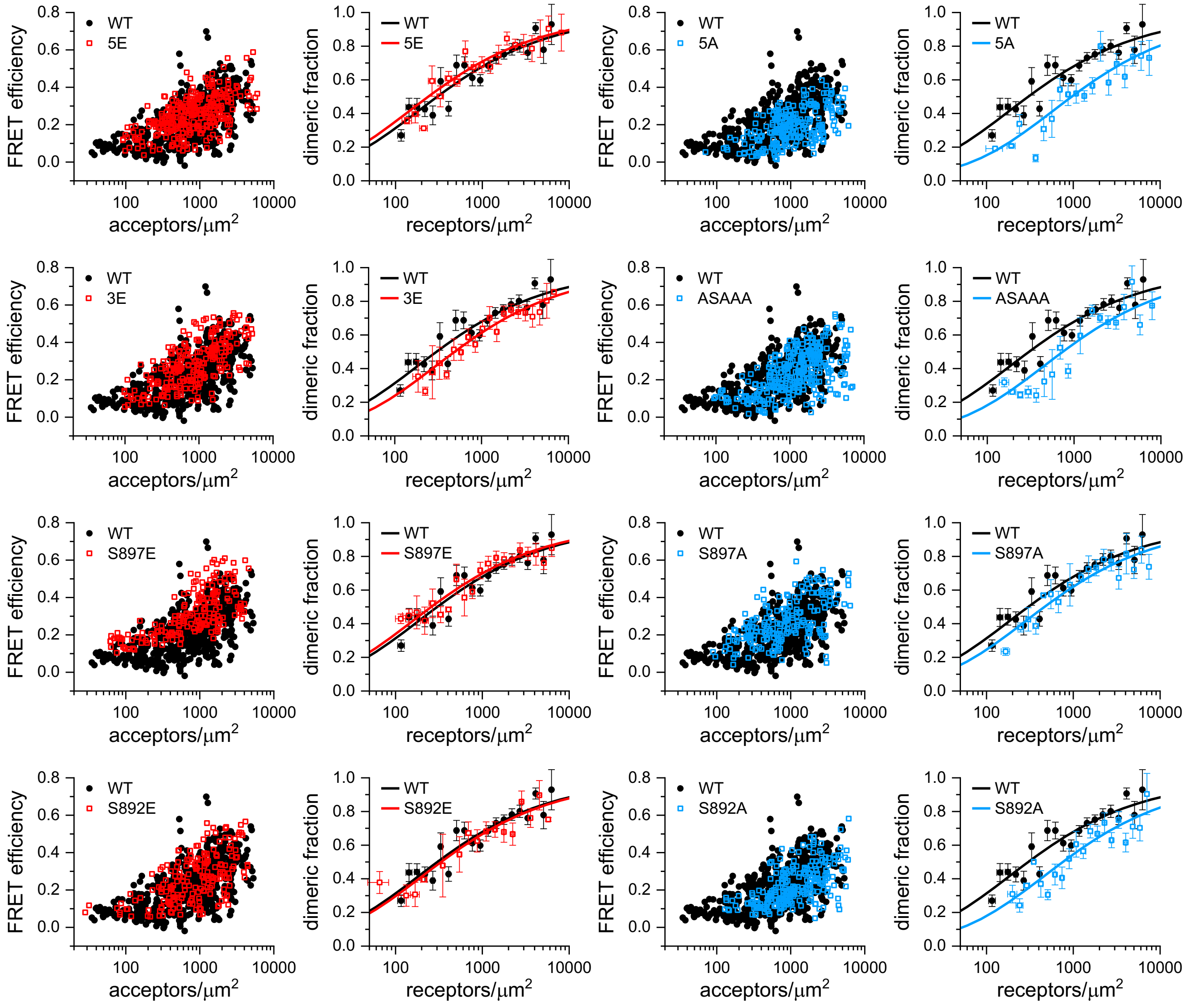

b

EphA2	K_{diss} (rec/μm²)
WT	302 ± 68
5E	238 ± 73*
3E	409 ± 144*
S897E	259 ± 63
S892E	334 ± 154
5A	958 ± 347*
ASAAA	698 ± 209*
S897A	465 ± 149*
S892A	756 ± 247*

Supplementary Figure 8. FSI-FRET analysis of dimerization for EphA2 kinase-SAM linker mutants. (**a**) FRET efficiencies, measured in individual cells, are shown as a function of EphA2-EYFP (acceptor) concentrations in the first and third columns. The data for the EphA2 phosphomimetic mutants (red symbols) and non-phosphorylatable mutants (blue symbols) are compared to EphA2 WT data (black symbols; from ref.[5]). These FRET efficiencies have been corrected as described[6] for "proximity FRET", which arises due to random proximity of donors and acceptors in the two-dimensional plasma membrane.

Best fit dimerization curves were obtained by fitting the FRET data with equation (2) in the Methods and are shown as solid lines in the second and fourth columns. The experimental data were binned and are shown as averages and standard errors. The number of data points, N, is as follows: WT (N=670), 5E (N=314), 3E (N=235), S897E (N=191), S892E (N=188), 5A (S892A/S897A/T898A/S899A/S901A; N=234), ASAAA (S892A/T898A/S899A/S901A; N=317), S897A (N=196), and S892A (N=241). (**b**) Best-fit parameters. K_{diss} is the two-dimensional dissociation constant. The errors are 68% confidence intervals from the fit. Statistical significance ($P<0.05$) for comparison with EphA2 WT was determined by one-way ANOVA followed by the Tukey's multiple comparisons test and is indicated with an asterisk (the exact P values are 0.057 for EphA2 S897E, 0.365 for EphA2 S892E and $P<0.0001$ for all other EphA2 mutants). $\tilde{E}$: Intrinsic FRET; *d:* average distance between the fluorescent proteins in the EphA2 dimers. See Methods for details. Source data are provided in the Source Data file.

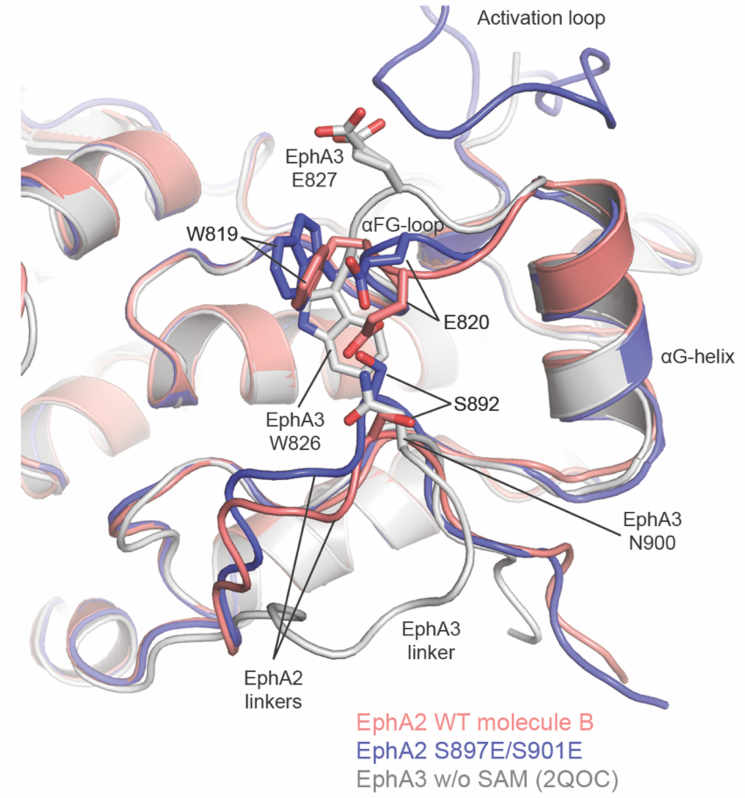

Supplementary Figure 9. EphA2 W819 and E820 as sensors of kinase-SAM linker phosphorylation. Overlay of the EphA2 WT kinase-SAM domain structure (molecule B), the EphA2 S897E/S901E mutant kinase-SAM domain structure, and the EphA3 kinase-linker structure (PDB 2QOC [http://doi.org/10.2210/pdb2QOC/pdb]); ref. [3]. In EphA2, E820 in the αFG-loop is positioned just above S892 and may act as a sensor for linker phosphorylation. The EphA2 αFG-loop, including W819, is part of an allosteric network that relays information about linker phosphorylation to the activation loop. The E827 sidechain is observed in two different conformations in the EphA3 crystal structure.

SUPPLEMENTARY REFERENCES

1. Klammer, M. et al. Phosphosignature predicts dasatinib response in non-small cell lung cancer. *Mol Cell Proteomics* **11**, 651-68 (2012).
2. Wei, Q. et al. A new autoinhibited kinase conformation reveals a salt-bridge switch in kinase activation. *Sci Rep* **6**, 28437 (2016).
3. Davis, T.L. et al. Autoregulation by the juxtamembrane region of the human ephrin receptor tyrosine kinase A3 (EphA3). *Structure* **16**, 873-84 (2008).
4. Troster, A. et al. NVP-BHG712: Effects of Regioisomers on the Affinity and Selectivity toward the EPHrin Family. *ChemMedChem* **13**, 1629-1633 (2018).
5. Gomez-Soler, M. et al. Engineering nanomolar peptide ligands that differentially modulate EphA2 receptor signaling. *J Biol Chem* **294**, 8791-8805 (2019).
6. King, C., Raicu, V. & Hristova, K. Understanding the FRET signatures of interacting membrane proteins. *J Biol Chem* **292**, 5291-5310 (2017).
