# Supplementary material for: Regulation of the EphA2 receptor intracellular region by phosphomimetic negative charges in the kinase-SAM linker
Source: Nat Commun. 2021 Dec 2;12:7047. doi: 10.1038/s41467-021-27343-z (PMC8639986; doi:10.1038/s41467-021-27343-z)
Supplement: Supplementary file 3 — Reporting Summary [file 41467_2021_27343_MOESM3_ESM.pdf]

nature research

Corresponding author(s): Elena Pasquale, Bernhard Lechtenberg

Last updated by author(s): Oct 24, 2021

Reporting Summary

Nature Research wishes to improve the reproducibility of the work that we publish. This form provides structure for consistency and transparency in reporting. For further information on Nature Research policies, see our Editorial Policies and the Editorial Policy Checklist.

Statistics

For all statistical analyses, confirm that the following items are present in the figure legend, table legend, main text, or Methods section.

n/a	Confirmed	
☐	☒	The exact sample size (*n*) for each experimental group/condition, given as a discrete number and unit of measurement
☐	☒	A statement on whether measurements were taken from distinct samples or whether the same sample was measured repeatedly
☐	☒	The statistical test(s) used AND whether they are one- or two-sided<br>*Only common tests should be described solely by name; describe more complex techniques in the Methods section.*
☒	☐	A description of all covariates tested
☒	☐	A description of any assumptions or corrections, such as tests of normality and adjustment for multiple comparisons
☐	☒	A full description of the statistical parameters including central tendency (e.g. means) or other basic estimates (e.g. regression coefficient) AND variation (e.g. standard deviation) or associated estimates of uncertainty (e.g. confidence intervals)
☐	☒	For null hypothesis testing, the test statistic (e.g. *F*, *t*, *r*) with confidence intervals, effect sizes, degrees of freedom and *P* value noted<br>*Give P values as exact values whenever suitable.*
☒	☐	For Bayesian analysis, information on the choice of priors and Markov chain Monte Carlo settings
☒	☐	For hierarchical and complex designs, identification of the appropriate level for tests and full reporting of outcomes
☐	☒	Estimates of effect sizes (e.g. Cohen's *d*, Pearson's *r*), indicating how they were calculated

Our web collection on statistics for biologists contains articles on many of the points above.

Software and code

Policy information about availability of computer code

Data collection	Rigaku CrystalClear 2.0
Data analysis	Graphpad Prism versions 7, 8 and 9, Mosflm 7.1.0, XDS (version January 10, 2014), aimless 0.3.11, Phaser 2.5.6, CCP4 (version 7.1.000), Phenix (version dev-3965), Refmac 5.8.0158, Coot 0.9 , PyMOL 2.3.4, ATSAS 3.0.3-1, ProteinLynx global server (PLGS) version 2.5, DynamX 3.0, DECA 1.14, Sedfit 12.52, ScatterBrain (V2.82), ASTRA (Version 7.3.1.9, Wyatt Technology), MATLAB version R2020a.

For manuscripts utilizing custom algorithms or software that are central to the research but not yet described in published literature, software must be made available to editors and reviewers. We strongly encourage code deposition in a community repository (e.g. GitHub). See the Nature Research guidelines for submitting code & software for further information.

Data

Policy information about availability of data

All manuscripts must include a data availability statement. This statement should provide the following information, where applicable:

- Accession codes, unique identifiers, or web links for publicly available datasets
- A list of figures that have associated raw data
- A description of any restrictions on data availability

Atomic structures and diffraction data generated in this study have been deposited in the Protein Data Bank (PDB) under accession codes 7KJA (EphA2 WT), 7KJB (EphA2 S897E/S901E) and 7KJC (EphA2 S901E). HDX-MS data have been deposited in the Mass Spectrometry Interactive Virtual Environment (MassIVE, massive.ucsd.edu) database with accession code MSV000086658 [https://doi.org/doi:10.25345/C5921C]. Kinase screening data are presented in Supplementary Table 3. All other data supporting the conclusions are available in the article and in the Source Data file provided with this paper. This study uses publicly available data from PhosphoSite Plus (https://www.phosphosite.org) and the PDB under accession codes: 2QOC [http://doi.org/10.2210/pdb2QOC/pdb], 3KKA [http://doi.org/10.2210/pdb3KKA/pdb], 4PDO [http://doi.org/10.2210/pdb4PDO/pdb], 5EK7 [http://doi.org/10.2210/pdb5EK7/pdb], 6FNG [http://doi.org/10.2210/

pdb6FNG/pdb] and 6Q7D [http://doi.org/10.2210/pdb6Q7D/pdb]. Source data are provided with this paper.

Field-specific reporting

Please select the one below that is the best fit for your research. If you are not sure, read the appropriate sections before making your selection.

☒ Life sciences ☐ Behavioural & social sciences ☐ Ecological, evolutionary & environmental sciences

For a reference copy of the document with all sections, see nature.com/documents/nr-reporting-summary-flat.pdf

Life sciences study design

All studies must disclose on these points even when the disclosure is negative.

Sample size	X-ray data were collected on single crystals until acceptable completeness and redundancy was reached.
Data exclusions	X-ray reflections were truncated based on CC1/2 statistics and <(I)/(sigma I)>.
Replication	X-ray data were collected from single crystals. SAXS data for EphA2 WT were collected twice with consistent results. All other SAXS data were collected from single samples. HDX-MS data for EphA2 WT and 5E mutant were collected twice (each with 3 successful technical replicates) with consistent results. HDX-MS data for the other EphA2 mutants were collected once with 3 successful technical replicates.
Randomization	5% of reflections for each diffraction dataset were randomly allocated by the CCP4 program from Rfree calculation.
Blinding	Blinding is not relevant to our study, since there are no experiments where investigator bias could affect measurements or data analysis.

Reporting for specific materials, systems and methods

We require information from authors about some types of materials, experimental systems and methods used in many studies. Here, indicate whether each material, system or method listed is relevant to your study. If you are not sure if a list item applies to your research, read the appropriate section before selecting a response.

Materials & experimental systems

n/a	Involved in the study	
☐	☒	Antibodies
☐	☒	Eukaryotic cell lines
☒	☐	Palaeontology and archaeology
☒	☐	Animals and other organisms
☒	☐	Human research participants
☒	☐	Clinical data
☒	☐	Dual use research of concern

Methods

n/a	Involved in the study	
☒	☐	ChIP-seq
☒	☐	Flow cytometry
☒	☐	MRI-based neuroimaging

Antibodies

Antibodies used	Antibodies used: EphA2 (rabbit mAb #6997, Cell Signaling Technology); EphA2 phosphoS897 (rabbit mAb #6347, Cell Signaling Technology); homemade affinity-purified rabbit antibodies recognizing the phosphorylated S892 motif; and homemade affinity-purified rabbit antibodies recognizing the phosphorylated S901 motif. Goat-anti-rabbit secondary antibodies conjugated to HRP (A16110; Invitrogen/ThermoFisher Scientific).
Validation	Antibodies recognizing EphA2 and the S897 phosphorylated motif were purchased from Cell Signaling Technology and we have validated them using the EphA2 S897A mutant and EphA2 overexpression. The EphA2 pS892 and pS901 phosphospecific antibodies were validated by showing that they recognize EphA2 WT by immunoblotting, with a stronger signal under conditions promoting kinase-SAM linker phosphorylation, but do not recognize EphA2 in which the Ser in the relevant phosphorylated motif is mutated to Ala (S892A or S901A). In addition, the phosphospecific antibodies were shown to label transiently transfected EphA2 WT but not to recognize the corresponding phosphorylated motif in the EphA1 kinase-SAM linker.

Eukaryotic cell lines

Policy information about cell lines

Cell line source(s)	The following cell lines were purchased from the American Type Culture Collection (Manassas, VA): PC3 androgen-independent prostate cancer (CRL-1435), A549, Calu-3 and H1648 lung adenocarcinoma (CCL-185, HTB-55 and CRL-5882 respectively), HCC1937 triple negative breast cancer (CRL-2336), SW626 colon adenocarcinoma (HTB-78), SKOV3 ovarian serous cystadenocarcinoma (HTB-77) and HEK293T human embryonic kidney (CRL-3216). The Mel-Juso melanoma cell line

was purchased from the DSMZ (ACC 74); the HOP62 lung adenocarcinoma cell line from the tumor/cell line repository of the Developmental Therapeutics Program, Division of Cancer Treatment and Diagnosis, National Cancer Institute. The BxPC3 pancreatic cancer cell line was kindly provided by P. Itkin-Ansari (Sanford Burnham Prebys Medical Discovery Institute), the Panc1 cell line by F. Levine (Sanford Burnham Prebys Medical Discovery Institute); the U87, T98G and U251-MG glioblastoma cell lines by W. Stallcup (Sanford Burnham Prebys Medical Discovery Institute); the BT549 breast cancer cell line by R. Maki (Sanford Burnham Prebys Medical Discovery Institute); the MDA-MB-468 breast cancer cell line by K. Vuori (Sanford Burnham Prebys Medical Discovery Institute); and the MDA-MB-231 breast cancer cell line by J. Smith (Sanford Burnham Prebys Medical Discovery Institute).

Authentication

The PC3, MDA-MB-231, BT549, H2009, T98G, U87 and U251-MG cell lines were authenticated after completion of the experiments and PANC-1 several passages before use in the experiments by performing short tandem repeat analysis on isolated genomic DNA with the GenePrint® 10 System (Promega), and peaks were analyzed using GeneMarker HID (Softgenetics). Allele calls were searched against short tandem repeat databases28. Other purchased cell lines were used after few passages to expand cells obtained from the vendor and thus not authenticated. BxPC3 and MDA-MB-468 were not authenticated.

Mycoplasma contamination

We periodically test cell lines to verify lack of Mycoplasma contamination. If cells test positive, they are not used for experiments and they are discarded.

Commonly misidentified lines
(See ICLAC register)

The only commonly misidentified cell line used was the U251 cell line, which was validated by STR profiling.
